# Supplementary material for: Tubulin response to intense nanosecond-scale electric field in molecular dynamics simulation
Source: Sci Rep. 2019 Jul 19;9:10477. doi: 10.1038/s41598-019-46636-4 (PMC6642143; doi:10.1038/s41598-019-46636-4)

Supplementary information 2 to

Tubulin response to intense nanosecond-scale electric field in molecular dynamics simulation

P. Marracino[1], D. Havelka[2], J. Průša[2], A. T. Ayoub[3], J. Tuszynski[4,5], M. Liberti[6], F. Apollonio[6], **M. Cifra**[2*]

[1] Rise Technology srl., San Martino di Lupari, Via Monte Bianco 18, 35018, Italy

[2] Institute of Photonics and Electronics of the Czech Academy of Sciences, Chaberská 57, 18200, Prague, Czech Republic (*cifra@ufe.cz)

[3]Medicinal Chemistry, Heliopolis University for Sustainable Development, 3 Cairo - Belbeis Desert Rd, Egypt

[4]Department of Physics, University of Alberta, 11560 University Avenue Edmonton, Alberta T6G 1Z2, Canada

[5] DIMEAS, Politecnico di Torino, 10129, Turin, Italy

[6] Department of Information Engineering, Electronics, and Telecommunications, Sapienza University of Rome, Via Eudossiana 18, 00184, Rome, Italy

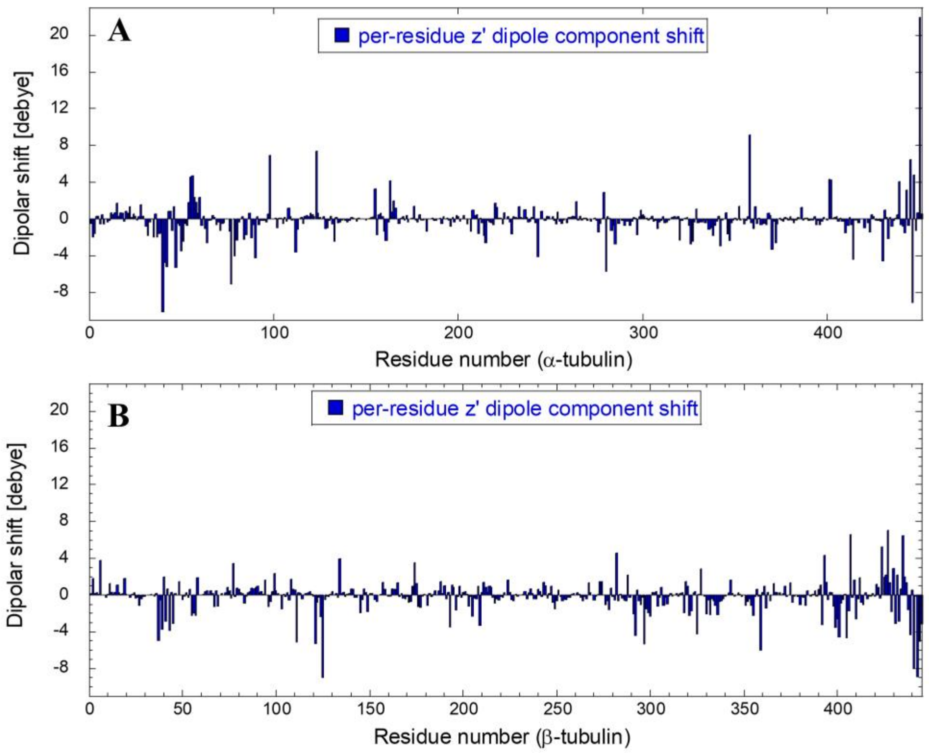

Supplement: Supplementary file 2 — S2 [file 41598_2019_46636_MOESM2_ESM.pdf]
