# Supplementary material for: Tubulin response to intense nanosecond-scale electric field in molecular dynamics simulation
Source: Sci Rep. 2019 Jul 19;9:10477. doi: 10.1038/s41598-019-46636-4 (PMC6642143; doi:10.1038/s41598-019-46636-4)
Supplement: Supplementary file 6 — S6 [file 41598_2019_46636_MOESM6_ESM.pdf]

Supplementary information 6 to

# Tubulin response to intense nanosecond-scale electric field in molecular dynamics simulation

P. Marracino[1], D. Havelka[2], J. Průša[2], A. T. Ayoub[3], J. Tuszynski[4,5], M. Liberti[6], F. Apollonio[6], **M. Cifra**[2*]

[1] Rise Technology srl., San Martino di Lupari, Via Monte Bianco 18, 35018, Italy

[2] Institute of Photonics and Electronics of the Czech Academy of Sciences, Chaberská 57, 18200, Prague, Czech Republic (*cifra@ufe.cz)

[3]Medicinal Chemistry, Heliopolis University for Sustainable Development, 3 Cairo - Belbeis Desert Rd, Egypt

[4]Department of Physics, University of Alberta, 11560 University Avenue Edmonton, Alberta T6G 1Z2, Canada

[5] DIMEAS, Politecnico di Torino, 10129, Turin, Italy

[6] Department of Information Engineering, Electronics, and Telecommunications, Sapienza University of Rome, Via Eudossiana 18, 00184, Rome, Italy

This supplementary information contains three DOI links with all scripts, software and trajectories

**1. https://dx.doi.org/10.5281/zenodo.2547671**

Molecular dynamics simulation trajectories of tubulin in electric field: traj_centered_no_field.xtc (no field), traj_centered_2_07.xtc (2e7 V/m)

all configuration and starting files, scripts and software

“Intial files” folder

The trajectories (link) have been performed starting from two equilibrated configurations, the first one obtained after 150 ns of simulation (conf_eq_regular_box.gro) for the regular simulation box (12x12x15 nm3), the second one after 30 ns of simulation (conf_eq_big_box.gro) for the big simulation box (12x12x30 nm3).

The simulation parameters are provided in the grompp.mdp file, together with the necessary topologies (topol_regular_box.top, topol_big_box.top, topol_Protein_chain_A.itp, topol_Protein_chain_B.itp, posre_Protein_chain_A.itp, posre_Protein_chain_B.itp)

Finally, the starting pdb file is also provided (1_TH.PDB).

“scripts and software” folder

All simulation were performed using Gromacs package version 4.6.5 (http://www.gromacs.org/Downloads_of_outdated_releases)

Data post-elaboration have been obtained by means of c++ codes (Code::Blocks v. 16.01, http://www.codeblocks.org/downloads/26) and Matlab (v. R2013a) built-in functions.

Secondary structures of protein have been calculated by means of the do_dssp program (https://swift.cmbi.umcn.nl/gv/dssp/ see attached file)

Representative scripts for Covariance Matrix method analysis are provided in:

- Cov_matrix_method.cpp
- Cov.m

A representative script for the calculation of residues dipoles is provided in:

- residues_dipoles.cpp

**2. https://dx.doi.org/10.5281/zenodo.2548247**

Molecular dynamics simulation trajectories of tubulin in electric field: traj_centered_07.xtc (1e7 V/m) and traj_centered_5_07.xtc (5e7 V/m)

**3. https://dx.doi.org/10.5281/zenodo.2548486**

Molecular dynamics simulation trajectories of tubulin in electric field: traj_centered_08.xtc (1e8 V/m), traj_centered_3_08.xtc (3e8 V/m), traj_ricentrata_2_08.xtc (2e8 V/m)
